# Effects of descending positive end-expiratory pressure on lung mechanics and aeration in healthy anaesthetized piglets

**DOI:** 10.1186/cc5030

**Published:** 2006-08-23

**Authors:** Alysson Roncally S Carvalho, Frederico C Jandre, Alexandre V Pino, Fernando A Bozza, Jorge I Salluh, Rosana S Rodrigues, João HN Soares, Antonio Giannella-Neto

**Affiliations:** 1 Biomedical Engineering Program, COPPE, Federal University of Rio de Janeiro, P.O. Box 68510, 21945-970, Rio de Janeiro, RJ, Brazil; 2 Electronic Engineering Department, Catholic University of Pelotas, Rua Félix da Cunha 412, 96010-000, Pelotas, RS, Brazil; 3 Clementino Fraga Filho Hospital, ICU, Federal University of Rio de Janeiro, Av. Brigadeiro Trompowsky, s/n°, 21950-900, Rio de Janeiro, RJ, Brazil; 4 National Institute of Cancer – 1, ICU, Praça Cruz Vermelha 23, 20230-130, Rio de Janeiro, RJ, Brazil; 5 Clementino Fraga Filho Hospital, Radiodiagnostic Service, Federal University of Rio de Janeiro, Av. Brigadeiro Trompowsky, s/n°, 21950-900, Rio de Janeiro, RJ, Brazil; 6 UNIGRANRIO, School of Veterinary Medicine, Rua Professor José de Sousa Herdy 1160, 25071-200, Duque de Caxias, RJ, Brazil

## Abstract

**Introduction:**

Atelectasis and distal airway closure are common clinical entities of general anaesthesia. These two phenomena are expected to reduce the ventilation of dependent lung regions and represent major causes of arterial oxygenation impairment in anaesthetic conditions. The behaviour of the elastance of the respiratory system (*E*_rs_), as well as the lung aeration assessed by computed tomography (CT) scan, was evaluated during a descendent positive end-expiratory pressure (PEEP) titration. This work sought to evaluate the potential usefulness of *E*_rs _monitoring to set the PEEP in order to prevent tidal recruitment and hyperinflation of healthy lungs under general anaesthesia.

**Methods:**

PEEP titration (from 16 to 0 cmH_2_O, tidal volume of 8 ml/kg) was performed, and at each PEEP, CT scans were obtained during end-expiratory and end-inspiratory pauses in six healthy, anaesthetized and paralyzed piglets. The distribution of lung aeration was determined and the tidal re-aeration was calculated as the difference between end-expiratory and end-inspiratory poorly aerated and normally aerated areas. Similarly, tidal hyperinflation was obtained as the difference between end-inspiratory and end-expiratory hyperinflated areas. *E*_rs _was estimated from the equation of motion of the respiratory system during all PEEP titration with the least-squares method.

**Results:**

Hyperinflated areas decreased from PEEP 16 to 0 cmH_2_O (ranges decreased from 24–62% to 1–7% at end-expiratory pauses and from 44–73% to 4–17% at end-inspiratory pauses) whereas normally aerated areas increased (from 30–66% to 72–83% at end-expiratory pauses and from 19–48% to 73–77% at end-inspiratory pauses). From 16 to 8 cmH_2_O, *E*_rs _decreased with a corresponding reduction in tidal hyperinflation. A flat minimum of *E*_rs _was observed from 8 to 4 cmH_2_O. For PEEP below 4 cmH_2_O, *E*_rs _increased in association with a rise in tidal re-aeration and a flat maximum of the normally aerated areas.

**Conclusion:**

In healthy piglets under a descending PEEP protocol, the PEEP at minimum *E*_rs _presented a compromise between maximizing normally aerated areas and minimizing tidal re-aeration and hyperinflation. High levels of PEEP, greater than 8 cmH_2_O, reduced tidal re-aeration but increased hyperinflation with a concomitant decrease in normally aerated areas.

## Introduction

It is well known that about 90% of the patients under general anaesthesia develop atelectasis and airway closure, mainly in dependent lung regions [1,2]. Muscle paralysis, which reduces the displacement of the diaphragm in dependent lung, results in atelectasis and airway closure in anaesthetized patients [3,4]. This effect is enhanced when large inspiratory fractions of oxygen are used during anaesthesia [2,5]. The anaesthesia-induced changes in pulmonary aeration are highly correlated with shunt as well as the decrease in the arterial oxygen tension, and also contribute to postoperative pulmonary complications such as pulmonary infection [2].

The use of recruitment manoeuvres has been proposed, to re-expand previously collapsed areas, with less deleterious effects than the institution of a positive end-expiratory pressure (PEEP) [2,6]. However, lung instability during general anaesthesia may require several recruitment manoeuvres, resulting in frequent derecruitment-recruitment episodes. Given that the required pressure to keep an airway or an alveolus open is lower than that required to recruit previously collapsed tissue, the administration of a PEEP subsequently to a recruitment manoeuvre may prevent atelectasis more effectively than just setting a PEEP without previous lung expansion. Simply performing a descending PEEP titration may have similar effects in healthy lungs, because lower pressures may be needed to open ventilatory units than those in diseased lungs.

Nonetheless, setting the PEEP is also difficult, because it should prevent cyclic derecruitment of alveoli or airways while keeping the lung open with less overdistension, thus avoiding tissue stress and damage induced by mechanical ventilation [7,8]. Focusing on respiratory system mechanical properties, the best PEEP may be recognized as the pressure for which the elastance of the respiratory system (*E*_rs_) is minimal during a PEEP titration manoeuvre. This approach has been suggested to be easily applicable to the clinical routine, especially in intensive care units [9].

In the present study, both the behaviour of *E*_rs _and the lung aeration assessed by computed tomography (CT) scan were evaluated in healthy anaesthetized and paralyzed piglets, during a descending PEEP titration manoeuvre, with a previous full lung re-aeration. This study sought to evaluate the potential usefulness of monitoring *E*_rs _to set the PEEP so as to prevent tidal recruitment and overdistension of healthy lungs under general anaesthesia. The correspondences and contrasts between *E*_rs _and the distributions of lung aeration, and particularly the distribution of lung aeration at the PEEP of minimum elastance, were examined.

## Materials and methods

### Ethical approval

The protocol was submitted to and approved by the local Ethics Commission for Assessment of Animal Use in Research (CEUA/FIOCRUZ).

### Animal preparation

Six mixed-breed female Landrace/Large White piglets (17 to 20 kg) were medicated with midazolam (Dormire; Cristália, São Paulo, Brazil) and subsequently intubated and connected to a mechanical ventilator in the supine position in spontaneous mode with a PEEP of 5 cmH_2_O and an inspiratory oxygen fraction (FiO_2_) of 1.0. A flexible catheter was introduced into the left femoral artery for continuous pressure monitoring (model 1290A; Hewlett-Packard, California, USA) and for blood gas analyses (I-STAT Corp, New Jersey, USA with EG7+ cartridges), to confirm the health status before the tests. The right femoral vein was also catheterized for drug administration. All animals were sedated with a continuous infusion of ketamine (Ketamina; Cristália, São Paulo, Brazil) delivered at a rate of 10 mg/kg per hour and paralysed with pancuronium (Pavulon; Organon Teknika, São Paulo, Brazil) at 2 mg/kg per hour. Invasive arterial blood pressure, electrocardiogram and peripheral oxygen saturation (CO2SMO; Dixtal, São Paulo, Brazil) were monitored continuously throughout the experiment. Respiratory mechanics was monitored with a purpose-built device. The opening airway pressure (*P*_aw_) was measured by a pressure transducer (163PC01D48; Honeywell Ltd, Illinois, USA) connected to the endotracheal tube, and flow was measured with a variable-orifice pneumotachometer (Hamilton Medical, Rhäzüns, Switzerland) connected to a pressure transducer (176PC07HD2; Honeywell Ltd, Illinois, USA). Both channels were amplified and filtered with fourth-order 33 Hz low-pass Butterworth analogue filters. *P*_aw_, flow and invasive arterial pressure were digitized into a personal computer running a program written in LabVIEW (National Instruments, Texas, USA). The sampling rate was 200 Hz per channel. The respiratory volume was calculated by numerical integration of the flow.

### Mechanical ventilation settings and PEEP titration procedure

All animals were ventilated with an Amadeus ventilator (Hamilton Medical, Rhäzüns, Switzerland) in controlled mandatory ventilation with a square flow waveform. The initial ventilator settings were FiO_2 _1.0, PEEP 5 cmH_2_O, tidal volume (*V*_T_) 8 ml/kg, inspiratory:expiratory ratio 1:2 and respiratory rate between 25 and 30 breaths per minute, to maintain normocapnia (arterial partial pressure of CO_2 _range 35 to 45 mmHg). On confirmation of the healthy lung status (arterial partial pressure of oxygen more than 500 mmHg), a PEEP titration was performed by decreasing PEEP from 16 cmH_2_O to 0 cmH_2_O in steps of 4 cmH_2_O, except from 8 cmH_2_O to 4 cmH_2_O where the steps were of 2 cmH_2_O. The time intervals between each step were 3 minutes, except at a PEEP of 16 cmH_2_O and zero end-expiratory pressure (ZEEP; 6 minutes each). All parameters were kept constant during the entire PEEP titration. At the end of the experiment the animals were killed with an intravenous injection of potassium chloride in the presence of deep sedation.

### CT scan procedure and image analysis

**Figure 1:**
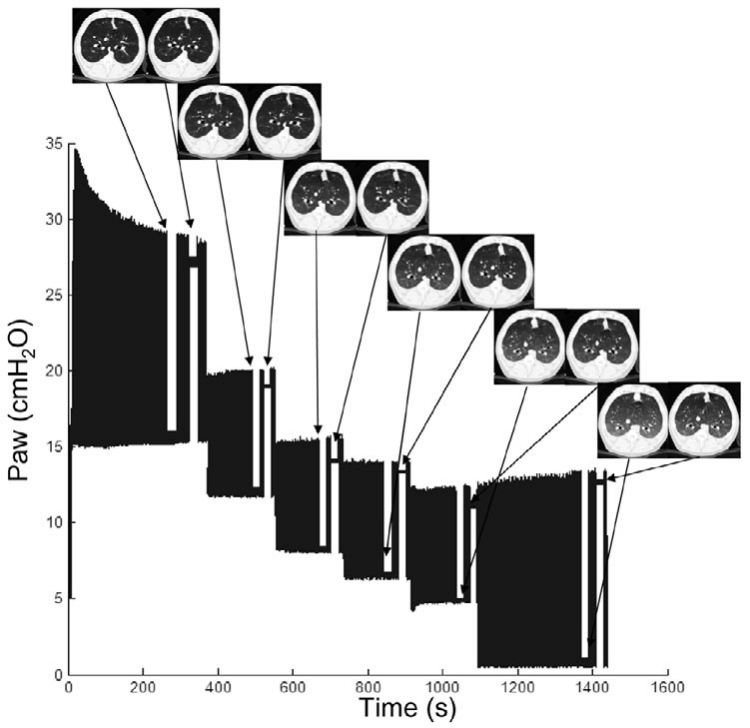
Time plot of airway pressure (*P*_aw_) during the positive end-expiratory pressure (PEEP) titration procedure. At the end of each PEEP step, a computed tomography (CT) scan was performed during end-expiratory and end-inspiratory pauses (CT scan images from a representative animal are shown).

Helical CT scans (Asteion, Toshiba, Tokyo, Japan) were obtained at a fixed anatomic level in the lower lobes of the lungs, caudal to the heart and cranial to the diaphragm in the supine position, corresponding to the largest transverse lung area. Each scan comprised five to seven thin-section slices (1 mm). The scanning time, tube current and voltage were 1 s, 120 mA and 140 kV, respectively. The actual image matrix was 512 × 512 and the voxel dimensions ranged from 0.22 to 0.29 mm. The scans were obtained at the end of each PEEP step, during end-expiratory and end-inspiratory pauses of 15 to 20 s (Figure 1).

The images were imported and analysed with a purpose-built routine written in MatLab (Mathworks). The lung contours, including the mediastinum, were traced manually to define the region of interest. The presence of hyperinflation (-1,000 to -900 Hounsfield units), normally aerated (-900 to -500 Hounsfield units), poorly aerated (-500 to -100 Hounsfield units) and non-aerated areas (-100 to +100 Hounsfield units) was determined, in accordance with the classification proposed by Gattinoni and colleagues [10] and Vieira and colleagues [11]. Furthermore, at each PEEP step the tidal re-aeration was calculated as the difference between end-expiratory and end-inspiratory poorly aerated and non-aerated areas [12]. Similarly, the tidal hyperinflation was obtained by the difference between end-inspiratory and end-expiratory hyperinflated areas [10].

To evaluate the cephalo-caudal gradient of aeration [13], a whole lung scan was performed during the PEEP titration manoeuvre at ZEEP in end-expiratory pause (one animal) and at a PEEP of 8 cmH_2_O in end-inspiratory pause (two animals). The CT scan adjustments were the same as described previously but with slices 1 mm thick, 10 mm apart from each other. Attenuation values outside the range -1,000 to +100, which contributed less than 2% of all counts, were excluded.

### Data analysis

The signals of *P*_aw_, flow and volume were used to obtain the parameters of the equation of motion of the respiratory system by least-squares linear regression, considering a linear single-compartment model (Equation 1):

*P*_aw _= *E*_rs _× *V*(*t*) + *R*_rs _× d*V*(*t*)/d*t *+ EEP     (1)

where *R*_rs _is the resistance of the respiratory system, *V*(*t*) is the volume, d*V*(*t*)/d*t *is the flow and EEP is the end-expiratory pressure. The regression analysis was performed in MatLab.

### Statistical analysis

Data are presented with median and range values, attributed to the respective PEEP values. The mechanical parameters (*E*_rs_, *R*_rs _and EEP) were calculated on a breath-by-breath basis from the last minute of each PEEP step, and immediately before the CT scans. The quality of fitting was assessed by the coefficient of determination of the regression. The peak and plateau pressures, as well as the applied PEEP values, were measured at each PEEP level. A Wilcoxon signed-rank test for paired samples was applied to compare changes in *E*_rs _for each PEEP step as well as changes in lung aeration between end-expiration and end-inspiration at each PEEP value. In all tests, *p *< 0.05 was considered significant.

## Results

The data on respiratory mechanics, the estimated elastance and resistance of the respiratory system and the estimated PEEP are presented in Table 1.

**Figure 2:**
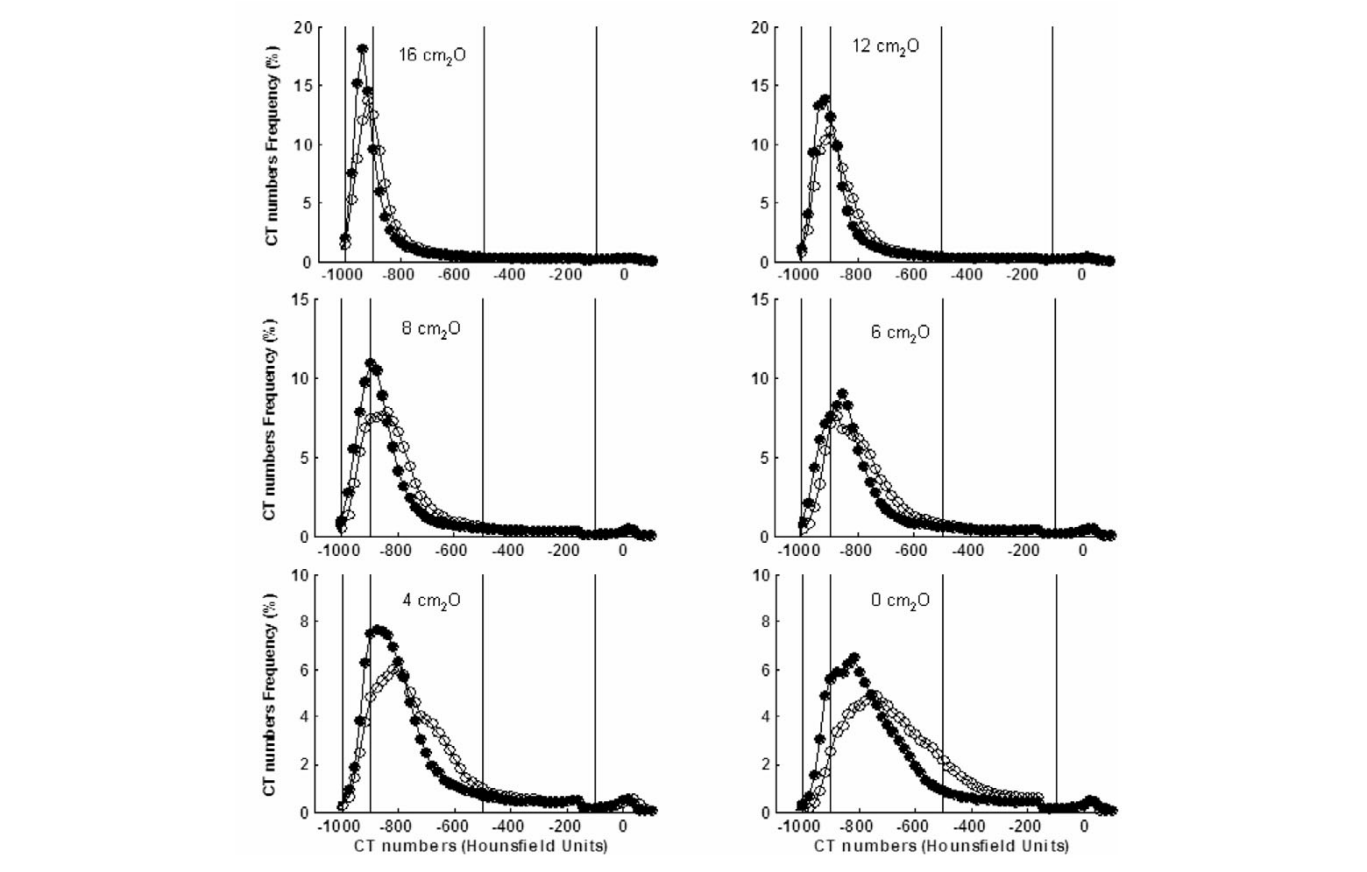
Median lung aeration distribution during positive end-expiratory pressure (PEEP) titration. Results are shown for all animals at end-expiratory (open circles) and end-inspiratory pauses (filled circles) during all PEEP titrations.

Figure 2 presents the dynamics of the distribution of lung aeration during PEEP titration for all animals, and depicts the average histograms of tissue densities, during the entire PEEP titration, at end-expiratory and end-inspiratory pauses. As can be seen from the graphs, the histograms always presented a unimodal distribution, and as PEEP decreased, the peak shifted to the right. The dynamics of the respiratory cycle resulted in a shift of the histogram from right to left for all levels of PEEP. Note that only at ZEEP it is possible to observe some poorly aerated areas that are re-aerated during inspiration.

### CT-scan morphological analyses and respiratory mechanics during PEEP titration

**Figure 3:**
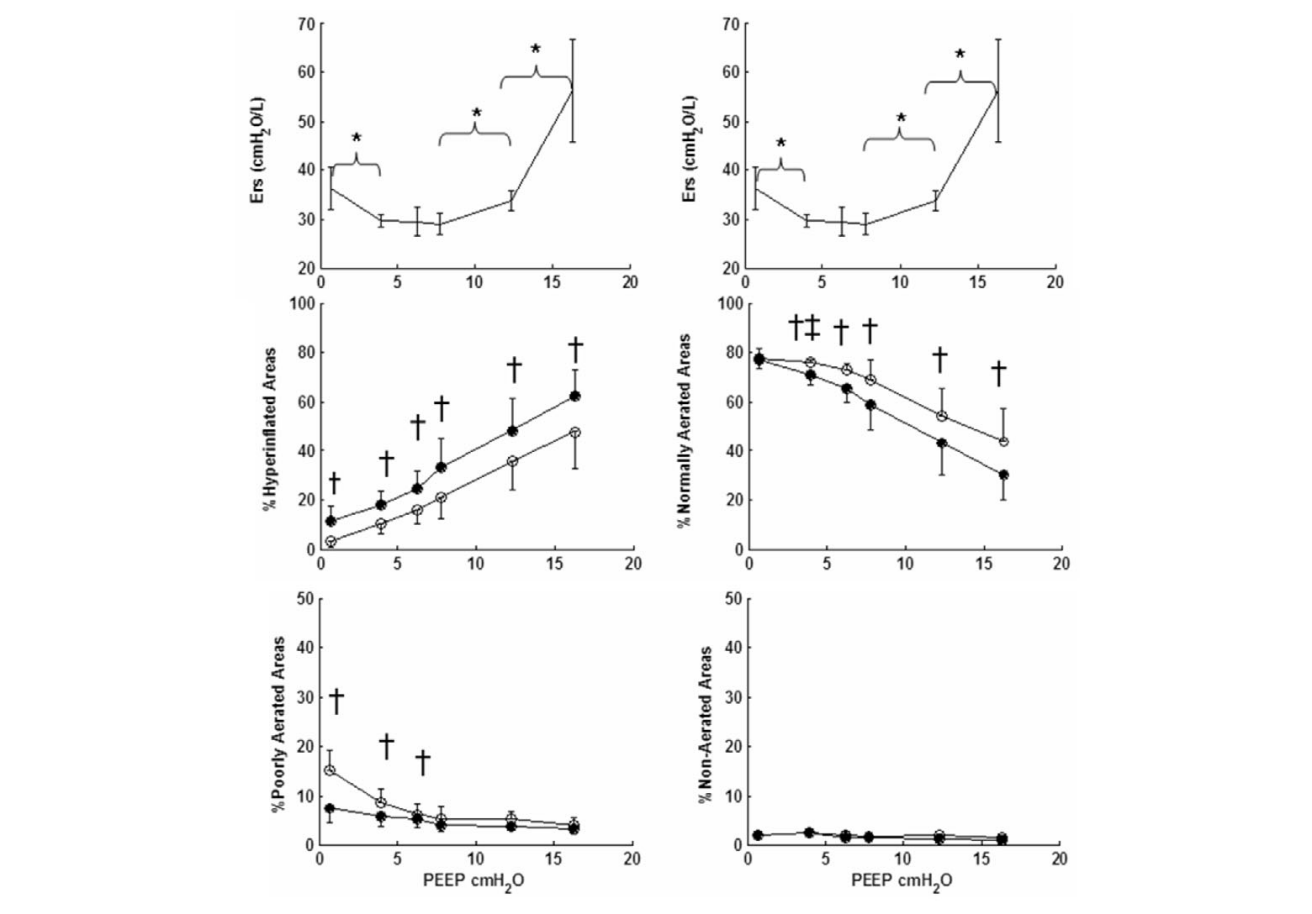
Comparative changes in *E*_rs_, and morphological analysis by computed tomography scan of the lung compartments. The open and filled circles indicate lung aeration changes at end-expiration and end-inspiration, respectively, and the bars represent the SD. Asterisks indicate a significant difference between the elastance of the respiratory system (*E*_rs_) for each positive end-expiratory pressure (PEEP) step (*p *< 0.05). Daggers indicate significant difference in lung aeration between end-expiration and end-inspiration at each PEEP (*p *< 0.05). Dagger and double dagger together indicate a non-significant difference (*p *= 0.065). The elastance plot is presented twice to allow comparisons between the elastance and the corresponding distribution of aeration.

The reduction of PEEP from 16 cmH_2_O to ZEEP resulted in a decrease in the hyperinflated areas (ranges decreased from 24–62% to 1–7% at end-expiratory pause and from 44–73% to 4–17% at end-inspiratory pause) while an increase in normally aerated areas was observed (from 30–66% to 72–83% at end-expiratory pause and from 19–48% to 73–77% at end-inspiratory pause). From 6 cmH_2_O to ZEEP, an increase in the poorly aerated areas was observed (from 3–9% to 10–21% at end-expiratory pause and from 3–7% to 5–13% at end-inspiratory pause) with no change in the non-aerated areas, which remained below 4% throughout the PEEP titrations (Figure 3). *E*_rs _and hyperinflated areas were highest at a PEEP of 16 cmH_2_O. As PEEP decreased, *E*_rs _reached a flat minimum between 8 and 4 cmH_2_O (non-significant difference in *E*_rs _in the range) and, at ZEEP, *E*_rs _had a value similar to that seen at a PEEP of 12 cmH_2_O (not significant).

**Figure 4:**
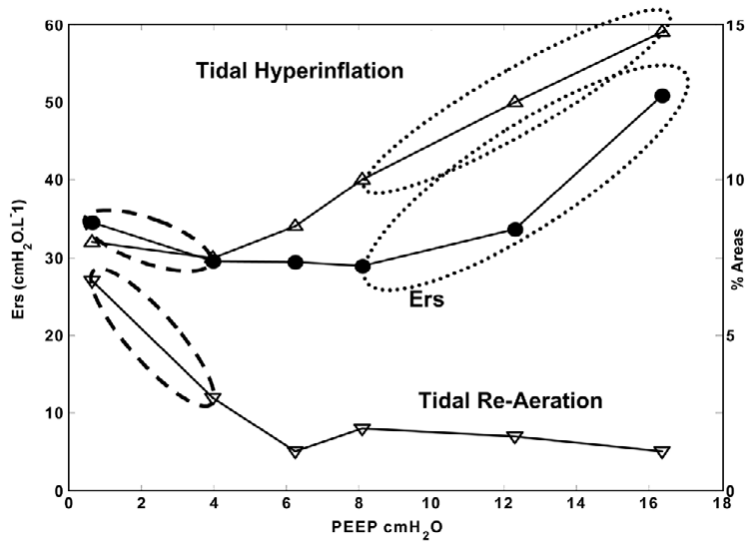
Elastance of the respiratory system, tidal re-aeration and tidal hyperinflation as a function of PEEP. Elastance of the respiratory system (*E*_rs_) is shown by filled circles, tidal re-aeration by downward triangles, and tidal hyperinflation by upward triangles. The dashed ellipses indicate the association between *E*_rs _and tidal recruitment growth for a positive end-expiratory pressure (PEEP) below 4 cmH_2_O. The dotted ellipses indicate the association between *E*_rs _and tidal hyperinflation growth at a PEEP of more than 8 cmH_2_O.

Figure 4 depicts the dynamics of tidal hyperinflation and re-aeration of *E*_rs _as a function of PEEP, during the PEEP titration manoeuvre.

**Figure 5:**
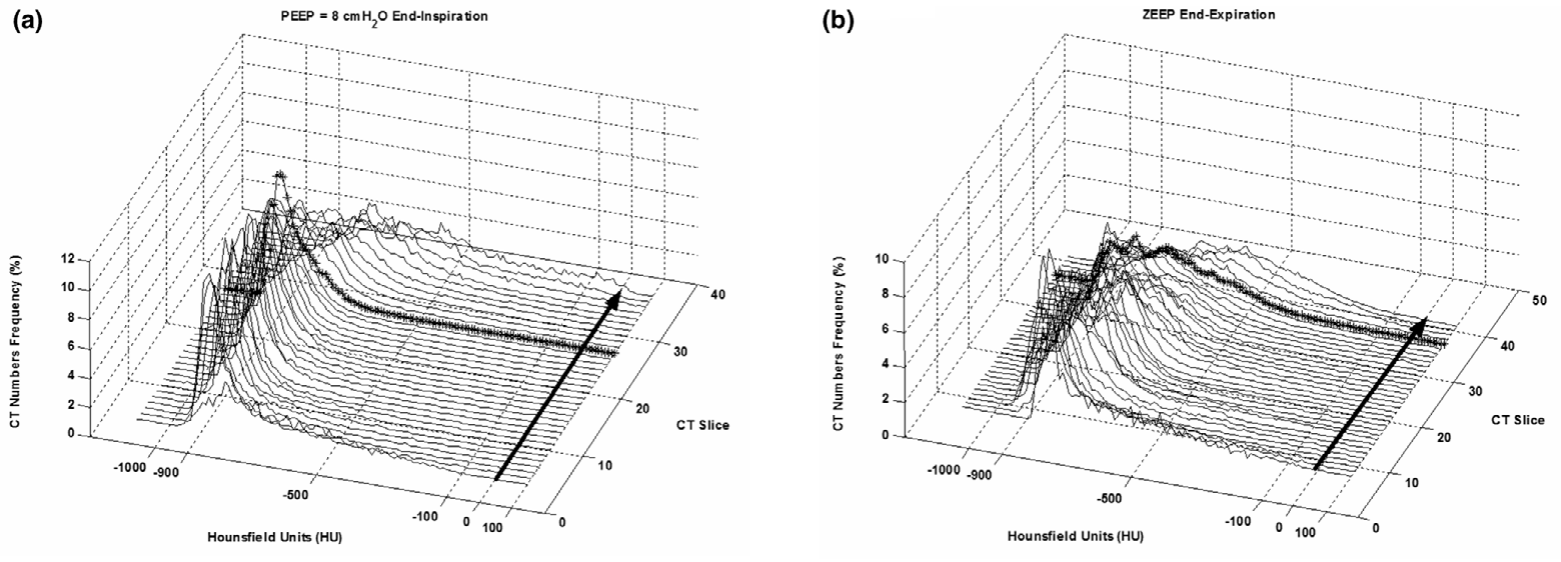
Aeration distribution assessed by whole-lung computed tomography (CT) scan in one animal. The arrow indicates the caudal portion. The CT scan slice level used in the present study is marked with crosses. Note that poorly aerated areas are more intense at zero end-expiratory pressure near the diaphragm (CT slices above 30 at panel (b) as compared to panel (a)).

Figure 5 depicts the whole-lung distribution of lung aeration assessed by CT scan in one of the studied animals during the PEEP titration. Each CT scan slice was obtained at a PEEP of 8 cmH_2_O (end-inspiratory pause; Figure 5a) and at ZEEP (end-expiratory pause; Figure 5b). Note that there are no cephalo-caudal gradients for the hyperinflated and normally aerated compartments. However, the poorly aerated areas are more intense at the diaphragmatic level (marked with crosses).

## Discussion

### Analysis of CT scans and elastic properties

The main objective of the present study was to evaluate the potential usefulness of *E*_rs _monitoring to set the PEEP so as to prevent tidal recruitment and hyperinflation of healthy lungs under general anaesthesia. It is clear that the descendent PEEP titration (measured with a *V*_T _of 7 to 9 ml/kg) promoted important changes in lung aeration distribution. In accordance with previous studies in healthy humans, the histograms of voxel distribution exhibited a unimodal pattern [14], and as PEEP decreased, the peak of the histogram shifted to the right, changing hyperinflated into normally aerated areas, and part of the latter into poorly aerated areas (Figure 2). High levels of PEEP (more than 8 cmH_2_O) resulted in a large hyperinflated area (greater than 30% on average). With a reduction in PEEP, the hyperinflated areas decreased with a consequent increase in normally aerated regions (Figure 3, top). Collapsed areas were never greater than 4% for any level of PEEP, and the poorly aerated areas increased only when PEEP fell below 6 cmH_2_O, becoming maximum at ZEEP (10 to 21%, during the end-expiratory pause).

Interestingly, the hyperinflated areas still appear at ZEEP (0 to 7% at end-expiration and 4 to 17% at end-inspiration). Very similar amounts of hyperinflated areas have been found by David and colleagues [15] using a dynamic CT scan technique in healthy piglets (weight 23 to 27 kg) mechanically ventilated with a PEEP ranging from 0 to 5 cmH_2_O and a *V*_T _of 12 ml/kg. Probably the supine position of the animals used in the present study resulted in a dorsal chest wall restriction, reducing the displacement of dependent regions with a concomitant hyperinflation in non-dependent lung areas. In fact, the hyperinflated areas appeared in non-dependent lung regions for PEEP values below 8 cmH_2_O.

Elastance behaved as expected with descending PEEP [16]. The *E*_rs _dynamics for all except one animal did not exhibit a sharp minimum as PEEP decreased. Nevertheless, a region of PEEP values (4 to 8 cmH_2_O; not significant) was found for minimal *E*_rs_. At these and lower PEEP values, the normally aerated areas became maximized and roughly flat, representing about 80% of the total selected area.

As observed in Figure 4, outside the PEEP of minimal *E*_rs_, the increased elastance seemed to correspond to changes in distinct ventilatory compartments. For PEEPvalues less than 4 cmH_2_O, *E*_rs _increased concomitantly with an increase in tidal re-aeration (from 3.5% at a PEEP of 4 cmH_2_O to 6.8% at ZEEP) and for PEEPvalues more than 8 cmH_2_O, *E*_rs _and tidal hyperinflation varied similarly to one another (from 8.0% at a PEEP of 8 cmH_2_O to 14.8% at 16 cmH_2_O). The physiological interpretation of these correspondences is straightforward: at low PEEP, *E*_rs _increases as a consequence of the derecruitment (and consequent tidal recruitment) of small airways and alveoli corresponding to the tidal re-aeration seen in the morphological analysis [17]; at high PEEP, *E*_rs _increases as a result of alveolar overdistension, reflected as alveolar tidal hyperinflation [11]. In the region of minimal *E*_rs_, the tidal re-aeration and hyperinflation areas possibly coexisted in balance, and this could explain the steady elastance [18]. It has already been reported elsewhere that normal lungs under general anaesthesia exhibit coexisting tidal re-aeration and hyperinflation at a large range of PEEP values [17].

Also interestingly, at ZEEP *E*_rs _increased to values similar to those observed at a PEEP of 12 cmH_2_O (Figure 3, left) and the CT images showed an increase in poorly aerated areas (reaching 15% of the region of interest); non-aerated areas remained close to zero. Such findings suggest an alternative interpretation of the areas classified as poorly aerated for normal lungs. It is known that each voxel contains hundreds of alveoli and its image represents an overall behaviour of all these units; consequently, a collective presence of non-aerated and aerated alveoli in the same voxel may decrease the gas:tissue ratio but not enough to indicate collapse [19]. In addition it seems unlikely, as suggested by Malbouisson and colleagues [12], that the tidal ventilation results in hyperdistension of normally aerated alveoli without the re-aeration of closed structures. Note in Figure 3 that at ZEEP the amount of normally aerated areas did not change during tidal inspiration, whereas poorly aerated areas decreased with a concomitant increase in hyperinflated areas. Possibly a part of poorly aerated areas became normally aerated whereas a similar amount of normally aerated areas became hyperinflated.

The contribution of chest wall elastance was not assessed in the present study. De Robertis and colleagues [20] suggested that the chest wall elastance of supine, anaesthetized and paralysed young piglets contributes significantly to *E*_rs _only at low volume or distending pressures. In view of this, it is possible that in the present study the increase in *E*_rs _at PEEP values less than 4 cmH_2_O might be partly attributed to the chest wall elastance. Nevertheless, during the six minute step at ZEEP, all animals presented a slow *E*_rs _increase that cannot be explained by changes in chest wall elastance and might be attributed to the lung component corresponding to the observed rise on tidal re-aeration (Figure 4). For high PEEP, the increase in *E*_rs _is exclusively attributed to the lung component [20] and seems to exhibit a particular correspondence to the magnification of hyperinflated areas.

According to the results presented in this study, for healthy lungs the institution of a PEEP based on *E*_rs _monitoring seems to correspond to the distribution of lung aeration assessed by CT scan. High levels of PEEP increase hyperinflated areas with a proportional decrease in normally aerated areas, resulting in mechanical stress to the lung parenchyma, which is probably reflected by the increase in *E*_rs_.

In humans, anaesthesia and paralysis are sufficient to produce non-aerated areas. These areas were negligible in the present study, but our results showed, at low PEEP, a progressive increase in poorly aerated areas and in *E*_rs_. The institution of PEEP seemed to re-aerate the poorly aerated areas at the expense of hyperinflating otherwise normally aerated areas, in non-dependent lung regions, suggesting that the hidden effect of PEEP is the overdistension of some alveoli. The biological cost of these procedures, tidal re-aeration at ZEEP or hyperinflation caused by the institution of a PEEP, was not assessed in the present study and remains an open question.

### Study limitations

The major limitation of this study is that the lung morphological analysis was based on a single slice of the CT scan taken at the juxta-diaphragmatic level. Reber and colleagues [16] offer data to support the choice of this slice level because the ventral–dorsal gradient seems to be more important than the diaphragm–carina gradient in healthy humans mechanically ventilated in the supine position during general anaesthesia. In fact, the CT scan slice near the juxta-diaphragmatic level, chosen in the present study as being representative of the whole lung, is likely to present histograms of densities similar to those of more apical portions of the lungs (Figure 5). Although the more caudal histograms skew more towards poorly and non-aerated areas than the others, they represent just a small amount of the total lung volume and thus possibly cause minor contributions to the overall ventilatory behaviour of the respiratory system.

The supine position is not physiological for the porcine model, and this could result in enhanced atelectasis [21]. However, in the present study the magnitude of non-aerated areas was always lower than 4%. Possibly the short duration of the protocol and the descendent PEEP strategy might explain these results.

The use of the present PEEP titration method can easily be applied under conditions of anaesthesia; however, as demonstrated by Suter and colleagues [22], the pressure of minimal *E*_rs _is dependent and increases with the magnitude of *V*_T_. A fixed small *V*_T _(such as 7 to 9 ml/kg) during the titration protocol is essential to minimize this effect and to prevent the adjustment of an inadequately low PEEP level.

The temporal effect on lung stability after a titration manoeuvre was not assessed in the present study. *E*_rs _may present slow dynamics until it converges to a stable value [16]. However, in normal lungs this time may be small, and in the present study it seemed to be achieved at the end of each PEEP step, especially for PEEP values ranging from 8 to 4 cmH_2_O. The need for recruitment manoeuvres after setting the PEEP at the minimum of the *E*_rs _was not assessed here; this might merit further study.

Pure oxygen was used in the present protocol, an atypical situation with regard to general anaesthesia. The fact that after 6 minutes of ventilation at ZEEP with pure oxygen the amount of non-aerated tissue was close to zero could be related to the limited time of exposure.

## Conclusion

In healthy piglets in the supine position, in a protocol of descendent PEEP, with a previous full lung re-aeration, the minimum respiratory system elastance corresponded to the greatest amount of normally aerated areas with approximately minimal tidal re-aeration and hyperinflation, according to morphologic analysis by CT scan. The *E*_rs _did not exhibit a sharp minimum and a range of PEEP from 4 to 8 cmH_2_O was found for minimal *E*_rs_. In comparison with ZEEP, the institution of this range of PEEP seemed to be a compromise to decrease the poorly aerated areas and tidal re-aeration as well as hyperinflation and tidal hyperinflation. Increased PEEP progressively enlarged the hyperinflated areas and tidal hyperinflation. These results could have implications for general anaesthesia management in healthy subjects, as far as gas exchange and/or potential ventilation-associated lung injury are concerned, and also for post-surgical and critical care.

## Key messages

• Atelectasis and intermittent closure of distal airways are common clinical occurrences during general anaesthesia.

• The administration of a PEEP titrated in a descent manoeuvre may prevent cyclic re-aeration.

• The PEEP at minimum *E*_rs _presented a compromise between maximizing normally aerated areas and minimizing tidal re-aeration and hyperinflation.

• High levels of PEEP, greater than 8 cmH_2_O, reduced tidal re-aeration but enlarged hyperinflation with an attendant decrease in normally aerated areas.

## Abbreviations

CT = computed tomography; EEP = end-expiratory pressure; *E*_rs _= elastance of the respiratory system; FiO_2 _= inspiratory oxygen fraction; *P*_aw _= opening airway pressure; PEEP = positive end-expiratory pressure; *R*_rs _= resistance of the respiratory system; *V*_T _= tidal volume; ZEEP = zero end-expiratory pressure.

## Competing interests

The authors declare that they have no competing interests.

## Authors' contributions

ARSC, FCJ, FAB, JHNS and JS performed the experiments. ARSC participated in the design of the study, performed the statistical analysis and wrote the manuscript. FCJ participated in the design of the study, discussed the results and revised the manuscript. AVP designed the experimental setup. FAB and JS participated in the design of the study and discussed the results. RR established the CT protocol and analysis. JHNS discussed the results. AG-N conceived and coordinated the study and helped to write the manuscript. All authors read and approved the final manuscript.

## Figures and Tables

**Table 1:** Respiratory mechanics data and regression parameters

	Descending PEEP titration steps					
	
PEEP_appl _(cmH_2_O)	16.4 (16.0–16.7)	12.5 (12.0–12.6)	8.3 (7.9–8.7)	6.3 (6–6.7)	4.1 (3.7–4.6)	0.8 (0.5–1.0)
*P*_peak _(cmH_2_O)	27.6 (24.4–31.3)	19.4 (18.8–20.6)	15.0 (13.5–17.8)	12.5 (11.4–13.1)	10.4 (9.6–11.2)	8.2 (6.9–10.4)
*P*_plateau _(cmH_2_O)	24.8 (22.5–28)	18.0 (17.4–19.4)	13.6 (12.3–15)	11.1 (10.3–11.8)	9.0 (8.4–9.8)	6.5 (5.6–7.5)
*E*_rs _(cm/l)	56.4 (41.7–71.9)	33.6 (30.5–36.8)	29.3 (26.2–32.0)	29.3 (25.0–34.6)	29.6 (27.2–31.6)	36.2 (30.4–42.6)
*R*_rs _(cmH_2_Ol^-1 ^s)	7.2 (5.3–8.4)	5.7 (4.9–6.9)	5.8 (5.3–7.0)	6.2 (5.4–7.7)	5.7 (5.3–8.1)	7.1 (6.3–10.1)
PEEP_est _(cmH_2_O)	16.3 (15.9–16.6)	12.3 (12–12.5)	8.1 (7.9–8.6)	6.2 (6.0–6.5)	4.0 (3.8–4)	0.7 (0.4–0.8)
*R*^2^	0.979 (0.968–0.983)	0.978 (0.974–0.982)	0.976 (0.964–0.976)	0.977 (0.964–0.979)	0.977 (0.969–0.979)	0.978 (0.970–0.982)

PEEP_appl_, applied positive end-expiratory pressure; *P*_peak_, peak ventilator pressure; *P*_platea_, plateau ventilator pressure; *E*_rs_, elastance of the respiratory system; *R*_rs_, resistance of the respiratory system; PEEP_est_, estimated positive end-expiratory pressure; *R*^2^, coefficient of determination of the regression analysis. Data are shown as medians and ranges.
